# Supplementary material for: Mutual inhibition between PTEN and PIP3 generates bistability for polarity in motile cells
Source: Nat Commun. 2018 Oct 26;9:4481. doi: 10.1038/s41467-018-06856-0 (PMC6203803; doi:10.1038/s41467-018-06856-0)
Supplement: Supplementary file 1 — Supplementary Information [file 41467_2018_6856_MOESM1_ESM.pdf]

SUPPLEMENTARY INFORMATION

for

Mutual Inhibition between PTEN and PIP3 Generates Bistability for Polarity in Motile Cells

Matsuoka and Ueda

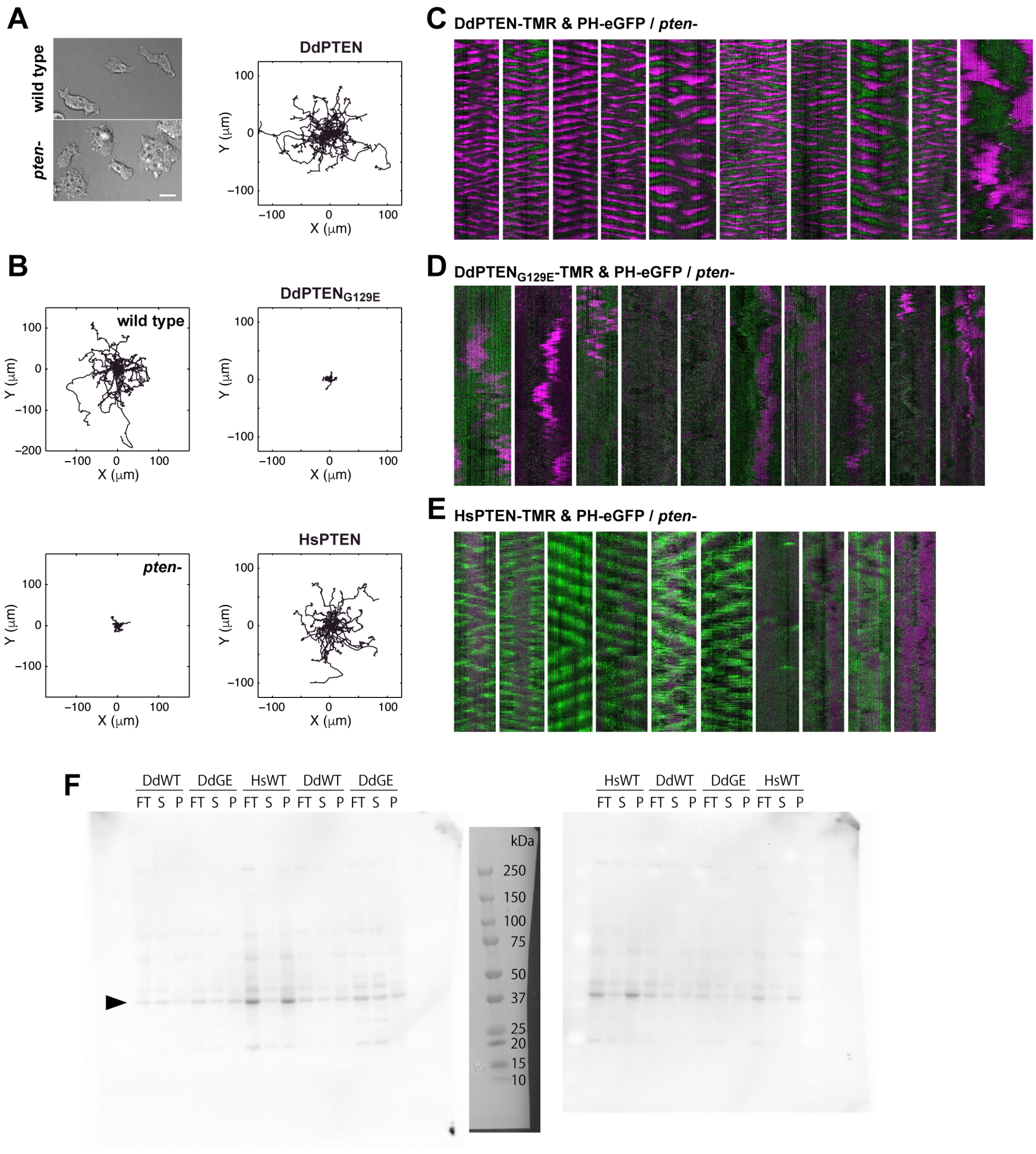

Supplementary Figure 1 Basal cell motility and polarity

(A) Cell Morphology. Scale bar, 10 μm. (B) Migration trajectories. Centroid tracks of 50 representative cells of wild-type (upper left), *pten*-null (lower left), *pten*-null expressing PH_{PKB}-eGFP and DdPTEN-Halo (upper right), $DdPTEN_{G129E}$-Halo (middle right) and HsPTEN-Halo (lower right) every 5 sec for 30 min are shown. (C-E) Gallery of kymographs. *pten*-null cells expressing DdPTEN-Halo (C), $DdPTEN_{G129E}$-Halo (D) or HsPTEN-Halo (E) and PH_{PKB}-eGFP were treated with 5 μM latrunculin A and 4 mM caffeine and observed under confocal microscopy at a time interval of 5 sec for 30 min. Fluorescence intensities measured along the cell periphery were arrayed against time. (F) Western blot images used for the quantification in Figure 1D. FT flow through, S supernatant, P pellet. Pellet fraction was concentrated 3-fold compared to the others.

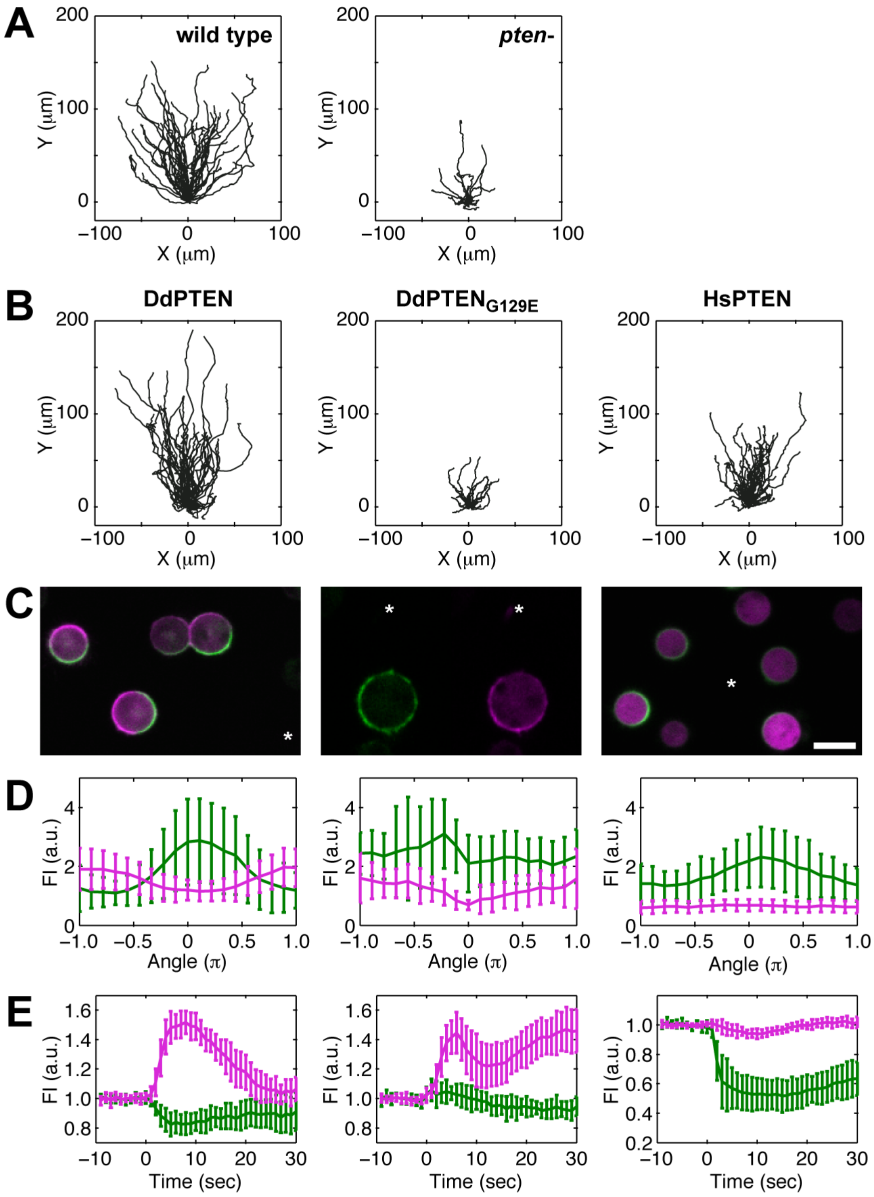

Supplementary Figure 2 Chemotactic response

(A,B) Migration trajectories. Centroid tracks of 50 representative cells every 5 sec for 20 min are shown. A micropipette containing cAMP solution was located on the *y*-axis (*x*=0, *y*>0). (C) Confocal images of latrunculin A-treated *pten*-null cells expressing DdPTEN-Halo (left), DdPTEN$_{G129E}$-Halo (middle) or HsPTEN-Halo (right) and PH$_{PKB}$-eGFP in response to the chemoattractant source placed at the position indicated by the asterisks. DdPTEN$_{G129E}$-Halo-expressing *pten*-null cells were treated with 40 μM LY294002. Scale bar, 10 μm. (D) Quantification of cells in (C). Fluorescence intensities of DdPTEN-Halo (*n*=29 cells; left), DdPTEN$_{G129E}$-Halo (*n*=11 cells; middle) or HsPTEN-Halo (*n*=34 cells; right) and PH$_{PKB}$-eGFP were measured along the cell periphery. The position facing the source was adjusted to angle=0. (E) The translocation of PTEN-Halo and PHD-GFP in response to step-wise uniform stimulation with 1 μM cAMP. Fluorescence intensities were measured in the cytoplasm every 1 sec (*n*=10 cells for all cell strains). Data are represented as mean ± SD.

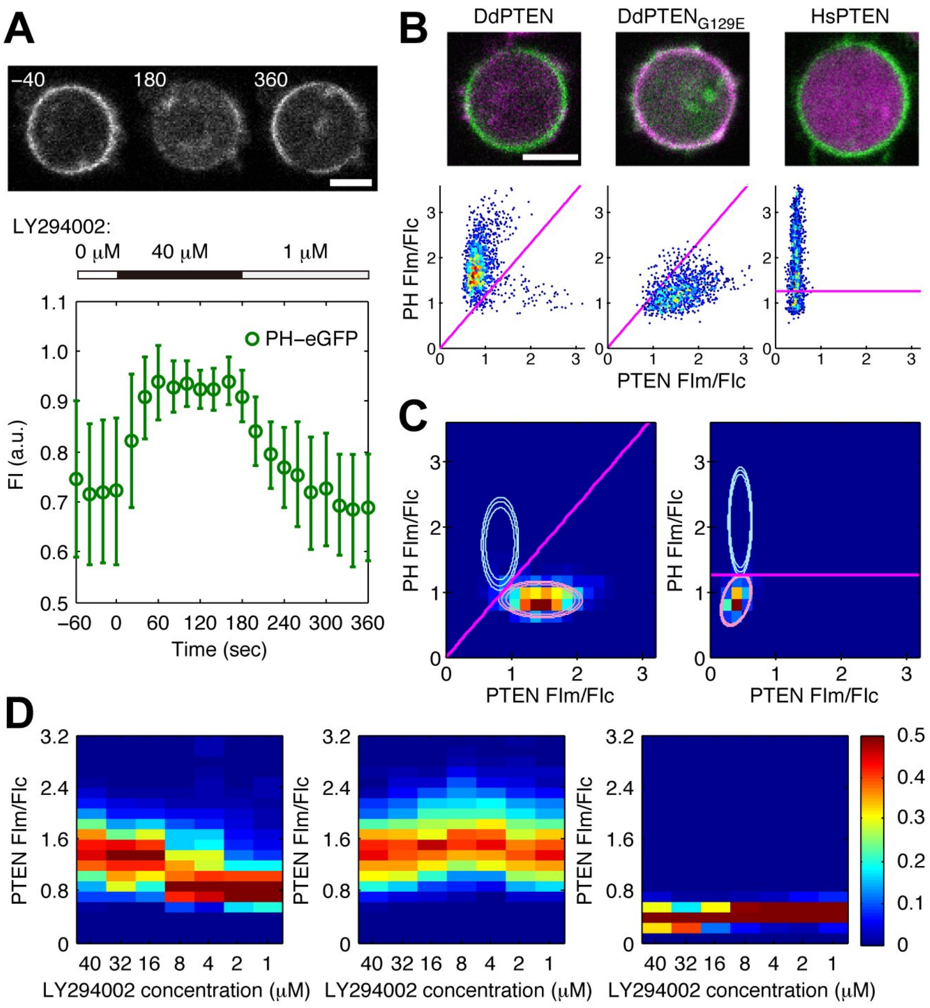

Supplementary Figure 3 Manipulation of PIP3 levels on the cell membrane

(A) (upper) Confocal images of the cells over-expressing myrPI3K2 and PH_{PKB}-eGFP in response to a transient increase in LY294002 concentration to 40 μM from t=0 to 180 sec. Time, sec. (lower) Quantification data. Fluorescence intensity of PH_{PKB}-eGFP was measured in the cytoplasm every 20 sec. Data are represented as mean ± SD (n=18 cells). (B) Representative cell images (upper) and heat scatter plots (lower) obtained from the cells shown in Figure 2 before treatment with LY294002. (C) Estimation of the border between two distributions of the fluorescence intensity plots in the PTEN-PIP3 plane at 1 μM and 40 μM LY294002. The heat scatter plots at 1 and 40 μM LY294002 shown in Figure 2B were fitted to two bivariate normal distributions with isodensity contours at probabilities of 0.95, 0.70 and 0.55 represented as solid lines for 1 μM (pale blue) and 40 μM (pink). The heat scatter plots at 40 μM LY294002 are shown as heat maps. The border was defined as a linear function so that it crosses the intercept of the isodensity contours (magenta line). The same analysis was performed for cells expressing DdPTEN (left) and HsPTEN (right). (D) Reciprocal PTEN membrane localization tightly coupled to the bistability of PIP3 enrichment. Heat maps showing the frequency of DdPTEN-Halo (left), $DdPTEN_{G129E}$-Halo (middle) and HsPTEN-Halo (right) intensities at the indicated LY294002 concentrations obtained from Figure 2B. Scale bars, 5 μm.

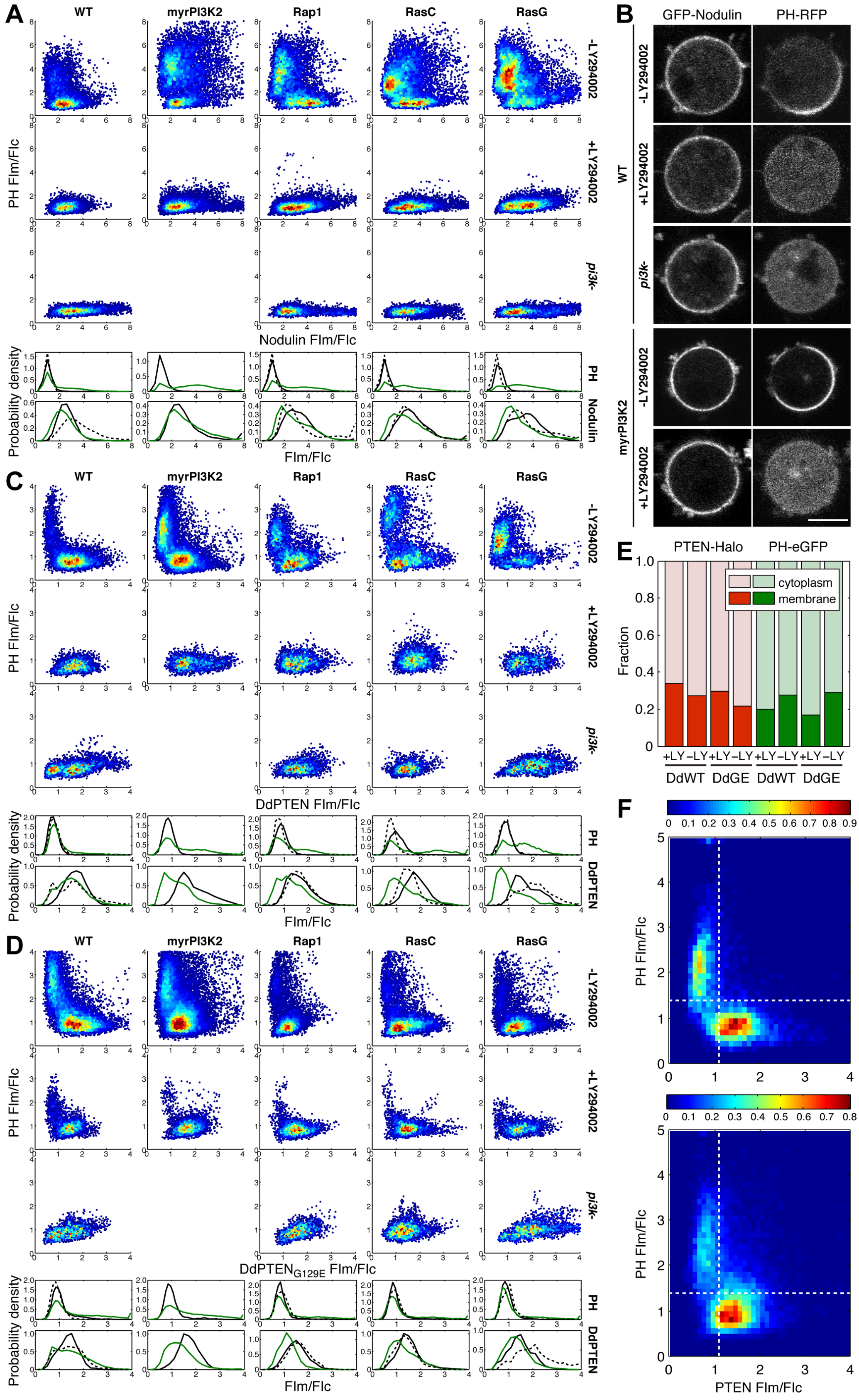

Supplementary Figure 4 Mutual inhibition between PIP3 and PTEN on the cell membrane

(A) Fluorescence intensities in a small ROI along the membrane shown as scatter plots in the [PIP2]-[PIP3] plane and histograms for [PIP2] and [PIP3] in the absence (green solid line) or presence (black solid line) of 40 μM LY294002 or in *pi3k*-null (dotted line) cells. *n*=21, 23, 25, 25, 26, 21, 27, 26, 25, 26, 22, 14, 20, 20 cells (from top left to bottom right). (B) Confocal images of living cells expressing GFP-Nodulin and PH_{PKB}-RFP. PI3K activity was enhanced by expressing myrPI3K2 and attenuated by treatment with 40 μM LY294002 or the deletion of 5 essential PI3K genes. (C) Scatter plots in the [DdPTEN]-[PIP3] plane and histograms of [PTEN] and [PIP3] at different PI3K activities. *n*=29, 44, 21, 12, 12, 11, 8, 8, 9, 5, 14, 9, 8, 9 cells. (D) Scatter plots in the [$DdPTEN_{G129E}$]-[PIP3] plane and histograms of [PTEN] and [PIP3] at different PI3K activities. *n*=37, 76, 27, 22, 26, 12, 12, 11, 9, 9, 9, 8, 12, 8 cells. (E) Biochemical quantification of PTEN or PIP3 amounts on the membrane. *D. discoideum* cells expressing either DdPTEN-Halo or $DdPTEN_{G129E}$-Halo in addition to PH_{PKB}-eGFP and myrPI3K2 were fractionated into membrane and supernatant fractions in the presence or absence of 40 μM LY294002 and analyzed by western blotting against anti-HaloTag antibody or anti-GFP antibody. (F) Heat maps showing the frequency of the simultaneous fluorescence intensities of DdPTEN-Halo (upper, *n*=44 cells) or $DdPTEN_{G129E}$-Halo (lower, *n*=76 cells) and PH_{PKB}-eGFP in a small ROI on the periphery of the cells expressing myrPI3K2. Higher frequencies are shown in hotter colors. x=1.100 and y=1.375 are shown in white dotted lines, and the probability of PTEN-Halo-TMR intensity larger than 1.100 and PH_{PKB}-eGFP intensity larger than 1.375 were 0.08 and 0.25 for DdPTEN and $DdPTEN_{G129E}$, respectively.

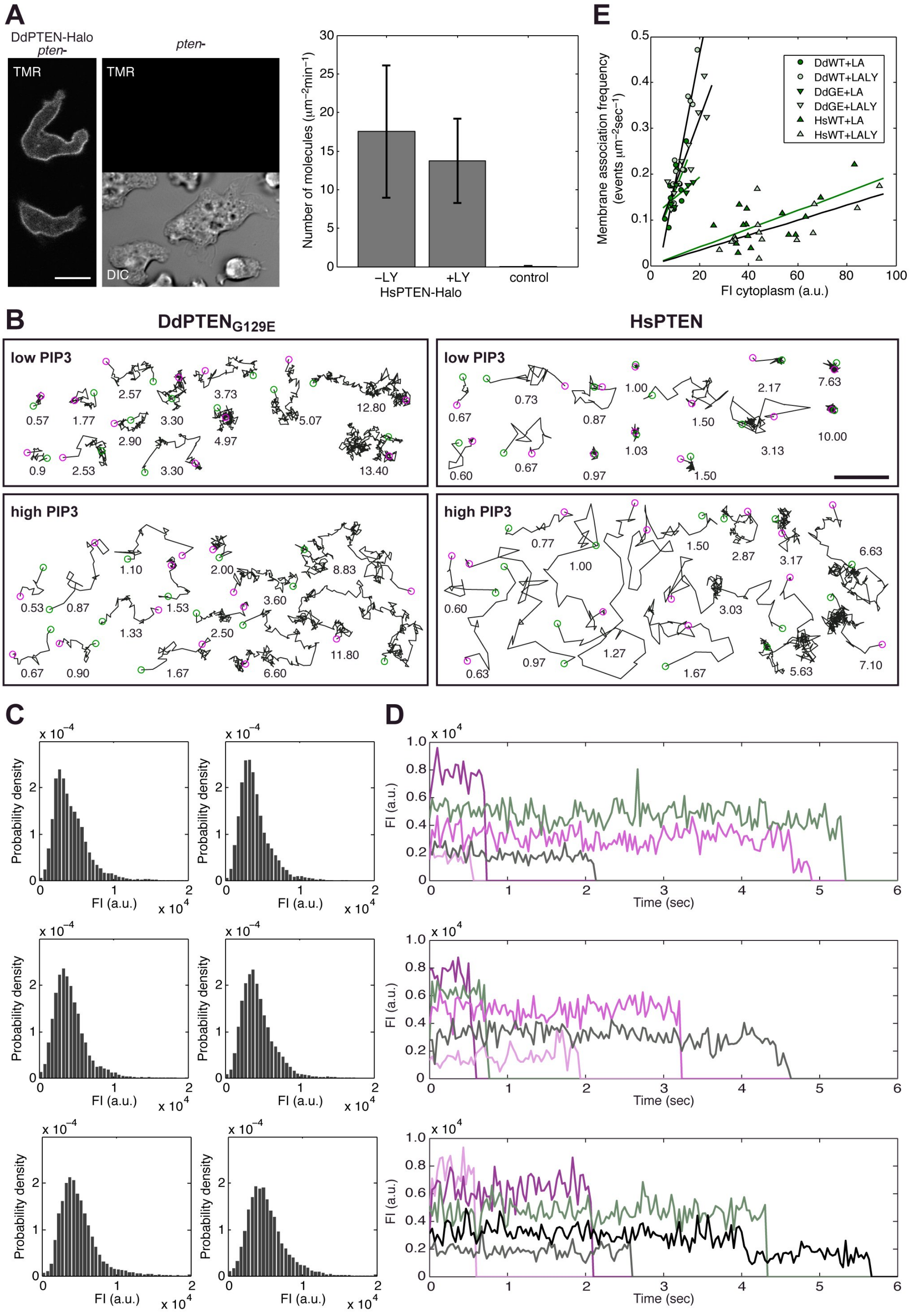

Supplementary Figure 5 Single-molecule imaging

(A) Negative controls for TMR-conjugated HaloTag ligand labeling. (left) *pten*-null cells either expressing

or not expressing DdPTEN-Halo observed under the same confocal microscopy after the same labling procedure. Scale bar, 10 μm. (right) Non-specific binding of TMR during the TIRFM observation was assessed by counting the number of fluorescent spots in wild-type cells expressing or not expressing either HsPTEN-Halo after the same labeling procedure. Data are shown as mean ± SD (*n*=4, 5 and 5 cells). *P*=0.0375 and 0.0067 (HsPTEN-LY versus control and HsPTEN+LY versus control) obtained by Welch's *t*-test. (B) Trajectories of single $DdPTEN_{G129E}$-Halo (left) and HsPTEN-Halo (right) molecules labeled with TMR undergoing lateral diffusion on the cell membrane of wild-type cells over-expressing myrPI3K2 in the presence (upper) or absence (lower) of LY294002. The start and end positions are indicated by magenta and green circles, respectively. Time, sec. Scale bar, 1 μm. (C) Single-peaked distributions of the fluorescence intensities of DdPTEN (top), $DdPTEN_{G129E}$ (middle) and HsPTEN (bottom) observed in the presence (left) or absence (right) of 40 μM LY294002. (D) Single-step photo-bleaching. Representative time series of single-molecule fluorescence intensities are shown for DdPTEN (top), $DdPTEN_{G129E}$ (middle) and HsPTEN (bottom). Most of the detected fluorescent spots exhibited single-step photo-bleaching, although some spots exhibited two-step photo-bleaching (black line in HsPTEN), suggesting the dimerization of PTEN. (E) Scatter plot showing the number of membrane association events counted in the cells with the indicated cytoplasmic fluorescence intensity. Solid lines show linear regression.

Supplementary Tables

Supplementary Table 1 Motility of *Dictyostelium* cells

	**Ax2**	***pten-***	**DdPTEN** ***pten-***	**DdPTEN$_{G129E}$** ***pten-***	**HsPTEN** ***pten-***
**Chemotactic motility**					
Velocity [μm/min]	6.21	3.19	6.99	2.89	5.15
Chemotactic index [a]	0.77	0.64	0.77	0.66	0.76
Number of cells	78	101	71	122	108
**Basal motility**					
Velocity [μm/min]	6.06	3.66	5.25	2.77	4.51
Number of cells	128	159	105	171	129

[a] Chemotactic index is the cosine of the angle formed by two sides between the start and end positions and the start and chemoattractant source positions.

Supplementary Table 2 Primers

Name	Sequence (5' to 3')
DdPTEN-KO1	ATGAGTAATTTATTAAGAGTTGC
DdPTEN-KO2	GTAATCATGGTCATAGCTGTTTCCTGCAGCTTACCAACGGATATATTGAATTG
DdPTEN-KO3	TTCTGCTTCAACCTTTGGAGCTGAATTTTC
DdPTEN-KO4	CACTGGCCGTCGTTTTACAACGTCGACAGTAAAGAACATAATATGAACG
PI3K2-1	CCAAAAGATCCATCACAAAGAAGAAGAAAAATGAGTGAAGGAATTATATC
PI3K2-2	GGGACGTCAAAAATGGGTTCATCAAAATCAAAACCAAAAGATCCATCACAAAG
PI3K2-3	CCACTAGTTTAATGAGCCCAACCATGGAAAATATC
DdPTEN-OE1	GGAGATCTAAAAATGAGTAATTTATTAAGAGTTG
DdPTEN-OE2	GGACTAGTTTCTGCTTCAACCTTTGGAGC
HsPTEN-OE1	GGGGATCCAAAAATGACAGCCATCATCAAAGAGATCG
HsPTEN-OE2	CCACTAGTTCCTCCGACTTTTGTAATTTGTGTATGC
