# Supplementary material for: Mutual inhibition between PTEN and PIP3 generates bistability for polarity in motile cells
Source: Nat Commun. 2018 Oct 26;9:4481. doi: 10.1038/s41467-018-06856-0 (PMC6203803; doi:10.1038/s41467-018-06856-0)
Supplement: Supplementary file 12 — Description of Additional Supplementary Files [file 41467_2018_6856_MOESM12_ESM.docx]

**Title:** Supplementary Movie 1.
**Description:** Chemotactic response of pten-null cells expressing DdPTEN-Halo and PHPKB-eGFP. A micropipette filled with 1 μM cAMP was positioned on the right side of the image. Scale bar, 10 μm. Time, min:sec.

**Title:** Supplementary Movie 2.
**Description:** Chemotactic response of a pten-null cell expressing DdPTENG129EHalo and PHPKB-eGFP. An asterisk indicates the location of the tip of a micropipette filled with 1 μM cAMP. Scale bar, 10 μm. Time, min:sec.

**Title:** Supplementary Movie 3.
**Description:** Chemotactic response of pten-null cells expressing HsPTEN-Halo and PHPKB-eGFP. An asterisk indicates the location of the tip of a micropipette filled with 1 μM cAMP. Scale bar, 10 μm. Time, min:sec.

**Title:** Supplementary Movie 4.
**Description:** Spontaneous dynamics of DdPTEN-Halo and PHPKB-eGFP in a ptennull cell in the presence of 5 μM latrunculin A and 4 mM caffeine, related to Figure 1. Scale bar, 2 μm. Time, min:sec.

**Title:** Supplementary Movie 5.
**Description:** Spontaneous dynamics of DdPTENG129E-Halo and PHPKB-eGFP in a pten-null cell in the presence of 5 μM latrunculin A and 4 mM caffeine, related to Figure 1. Scale bar, 2 μm. Time, min:sec.

**Title:** Supplementary Movie 6.
**Description:** Spontaneous dynamics of HsPTEN-Halo and PHPKB-eGFP in a ptennull cell in the presence of 5 μM latrunculin A and 4 mM caffeine, related to Figure 1. Scale bar, 2 μm. Time, min:sec.

**Title:** Supplementary Movie 7.
**Description:** Single molecules of DdPTEN-Halo labeled with TMR in myrPI3K2- overexpressing cells in the presence (left) or absence (right) of 40 μM LY294002, related to Figure 4. Scale bar, 3 μm. Time, sec.

**Title:** Supplementary Movie 8.
**Description:** Single molecules of DdPTENG129E-Halo labeled with TMR in myrPI3K2-overexpressing cells in the presence (left) or absence (right) of 40 μM LY294002, related to Figure 4. Scale bar, 3 μm. Time, sec.

Supplementary Movie 9.
**Description:**Single molecules of HsPTEN-Halo labeled with TMR in myrPI3K2-overexpressing cells in the presence (left) or absence (right) of 40 μM LY294002, related to Figure 4. Scale bar, 3 μm. Time, sec.
